# Lipoprotein(a) in calcific aortic stenosis: mechanistic insights, clinical evidence, and emerging therapies

**DOI:** 10.3389/fcvm.2026.1963618

**Published:** 2026-10-21

**Authors:** Wang Li, Shao Yun Shuang, You Bei An, Xu Yao, Wang Hao, Xu Xing Sheng, Pang Xue Lian, Meng Qing Feng

**Affiliations:** Department of Cardiology, Qilu Hospital (Qingdao), Shandong University, Qingdao, China

**Keywords:** autotaxin, calcific aortic stenosis, lipoprotein(a), oxidized phospholipids, pelacarsen, valve calcification

## Abstract

Calcific aortic stenosis (CAVS) is the most prevalent valvular heart disease affecting older adults, yet no validated pharmacological intervention can halt its progressive deterioration. Lipoprotein(a) [Lp(a)] denotes a genetically regulated low-density lipoprotein (LDL)-like particle, in which apolipoprotein(a) forms a covalent bond with apolipoprotein B-100. Cumulative genetic, epidemiological, imaging and mechanistic research supports a causal role for elevated circulating Lp(a) in the initiation, and suggests a contribution to the advancement, of calcific aortic valve lesions. Lp(a) mediates valve injury mainly via preferentially transporting oxidized phospholipids (OxPL), anchoring within aortic valve leaflets, triggering endothelial activation and inflammatory cascades, as well as stimulating the autotaxin–lysophosphatidic acid (ATX–LPA) axis; collectively these signals drive osteogenic differentiation and mineralization of valve interstitial cells (VICs). Higher Lp(a) levels correlate with incident aortic valve calcification, accelerated hemodynamic deterioration, augmented calcium deposition quantified by computed tomography (CT), and elevated risks of aortic valve replacement (AVR) or all-cause cardiac death. Conventional lipid-lowering agents fail to reverse disease trajectory in patients with established CAVS. Though proprotein convertase subtilisin/kexin type 9 (PCSK9) inhibitors only modestly cut Lp(a) concentrations, exploratory analyses have delivered preliminary suggestive signals for cardiovascular protection. RNA-targeted therapeutics and oral agents blocking Lp(a) assembly can reduce circulating Lp(a) by 70% up to over 95%, as demonstrated in early- and mid-phase clinical trials. Most notably, the ongoing Lp(a)FRONTIERS CAVS randomized trial is directly testing whether pelacarsen can decelerate CAVS progression, and no completed trial has yet demonstrated that Lp(a) lowering modifies CAVS disease. This narrative review summarizes Lp(a) biological characteristics, causal evidence linking Lp(a) to CAVS, together with opportunities and unresolved uncertainties surrounding Lp(a)-targeted therapeutic strategies.

## 1 Introduction

Calcific aortic stenosis (CAVS) represents a progressive fibrocalcific disorder characterized by aortic leaflet thickening, interstitial fibrosis and tissue mineralization. Once severe symptomatic stenosis emerges, surgical or transcatheter aortic valve replacement (TAVR) remains the sole intervention capable of improving long-term patient prognosis (1–3). Despite remarkable advancements in transcatheter and surgical valve interventions, no pharmacotherapy has been formally proven to prevent CAVS onset or reliably slow hemodynamic worsening after lesion formation (2–5). This unmet clinical demand gains growing significance as population aging expands the global burden of aortic valve disorders.

CAVS pathogenesis relies on active biological remodeling rather than passive degenerative wear of valve tissue. Endothelial injury, lipid infiltration, inflammatory cell recruitment, extracellular matrix (ECM) remodeling, fibrosis and osteogenic signaling constitute a protracted priming phase that precedes clinically detectable stenosis by decades (3–5). Large-scale genetic analyses and Mendelian randomization studies identified the LPA gene locus and genetically elevated Lp(a) as strong heritable risk determinants for aortic valve calcification and obstructive stenosis (6–9). Such landmark observations redirected research focus toward Lp(a) as a genetically supported causal risk factor and a potentially modifiable therapeutic target.

Lp(a) consists of apolipoprotein B-100-containing lipoprotein core, distinguishable from LDL via covalently bound apolipoprotein(a) [apo(a)]. Its plasma concentrations are predominantly governed by genetic variants and show substantial interindividual and interethnic heterogeneity (9–11). Given that Lp(a) acts as the primary carrier of circulating OxPL in human plasma, it simultaneously triggers lipid deposition, pro-inflammatory responses and pro-calcific bioactivity within valve tissue (12, 13). The present review integrates up-to-date evidence regarding Lp(a) biology, molecular mechanisms underlying valvular damage, clinical correlations with CAVS, and novel therapeutic pipelines targeting Lp(a).

We conducted a narrative review of the published literature on lipoprotein(a) [Lp(a)] and calcific aortic stenosis (CAVS). PubMed/MEDLINE was searched up to 25 July 2026 using combinations of the terms “lipoprotein(a)”, “Lp(a)”, “apolipoprotein(a)”, “calcific aortic valve stenosis”, “calcific aortic valve disease”, “aortic valve calcification”, “oxidized phospholipids”, “autotaxin”, “lysophosphatidic acid”, and relevant therapeutic agent names (pelacarsen, olpasiran, lepodisiran, zerlasiran, muvalaplin, and proprotein convertase subtilisin/kexin type 9 inhibitors). Reference lists of retrieved articles and recent reviews were additionally screened for relevant publications. Given the rapidly evolving therapeutic landscape, priority was given to landmark randomized trials, prospective cohort studies, Mendelian randomization studies and recent meta-analyses, and the evidence base was synthesized narratively rather than through a formal systematic search. This article is therefore a narrative review rather than a systematic review, and the search process was not preregistered.

## 2 Biology, epidemiology, and measurement of Lp(a)

Lp(a) features an LDL-like core carrying apolipoprotein B-100, linked to apo(a) through disulfide bonds. Apo(a) contains repetitive kringle IV domains; variable copy numbers of kringle IV type 2 repeats generate wide heterogeneity in apo(a) isoform size. In general, smaller apo(a) isoforms correspond to higher baseline circulating Lp(a) levels (9–11). Overwhelming interindividual variability of Lp(a) levels originates from variants within the LPA gene, whereas age, dietary habits and routine lifestyle modifications exert relatively marginal impacts on circulating concentrations (9–11). Selected clinical conditions including chronic kidney disease (CKD), chronic inflammatory disorders, hepatic dysfunction and hormonal fluctuations may alter measured Lp(a) values in specific patient subgroups (9, 10).

Approximately one-fifth of the global population exhibits Lp(a) levels exceeding commonly accepted high-risk cutoffs, yet the concentration distribution varies markedly across ethnic ancestries (9–11). Lp(a) levels are highest among Black populations and intermediate in Hispanic and South Asian groups, whereas they are lowest among East Asian and White populations (9, 10). Because Lp(a) is largely genetically determined, this heterogeneity is driven primarily by variants within the LPA gene and by apo(a) isoform size: in general, smaller apo(a) isoforms are associated with higher circulating Lp(a) concentrations, whereas larger isoforms produce lower levels (9–11). Across the ancestries studied, the association between higher Lp(a) and aortic valve calcification appears consistent, as reported in both White and Black participants of the Multi-Ethnic Study of Atherosclerosis (14) and synthesized in the European Atherosclerosis Society consensus statement (10); however, evidence in other ancestries remains more limited, underscoring the need for multi-ethnic recruitment in future trials. Contemporary lipid management guidelines recommend one-time Lp(a) testing for all adults, preferably utilizing isoform-insensitive detection assays (9–11). Laboratory results should be reported with assay-matched calibration units; direct mathematical conversion between mass units (mg/dL) and molar units (nmol/L) is discouraged due to variable apo(a) isoform sizes across individuals (9–11). This reflects a practical limitation: isoform-sensitive assays report particle mass and are influenced by apo(a) isoform size, whereas isoform-insensitive assays report molar concentration, and the conversion factor between the two varies across assays and laboratories (9–11). Consequently, reported Lp(a) values are not directly interchangeable across assay platforms, which represents an important source of the between-study heterogeneity discussed later in this review (9–11, 15, 16). Clinicians may adopt a practical risk stratification standard: concentrations <30 mg/dL (<75 nmol/L) correspond to low cardiovascular risk, 30–50 mg/dL (75–125 nmol/L) represent intermediate risk, and values ≥50 mg/dL (≥125 nmol/L) indicate clinically meaningful Lp(a) elevation (11).

In patients diagnosed with CAVS, Lp(a) testing delivers prognostic information independent of conventional LDL cholesterol metrics. Elevated Lp(a) can persist even under well-controlled LDL-C levels, and such patients carry a genetic predisposition to earlier valve calcification or accelerated disease progression (6–9, 14–19). Nevertheless, Lp(a) measurement cannot serve as an isolated diagnostic biomarker for CAVS at present, and its value for guiding echocardiographic surveillance intervals remains to be prospectively validated (2, 9–11).

## 3 Mechanistic links between Lp(a) and calcific aortic valve disease

### 3.1 Valvular retention and oxidized phospholipid signaling

Maximum mechanical shear stress concentrates on the aortic surface of valve leaflets, where endothelial disruption facilitates infiltration and retention of apoB-bearing lipoproteins (4, 5). Lp(a) occupies a unique pathogenic position as the dominant carrier of systemic OxPL within human plasma (12, 13). OxPL activates valvular endothelial cells, promotes monocyte chemotaxis, amplifies pro-inflammatory cytokine secretion and stimulates proliferative signaling in VICs (13, 20–22). Among patients with mild-to-moderate CAVS, higher Lp(a) and OxPL-apoB levels correlate with steeper increases in peak aortic jet velocity, alongside elevated risks of AVR or cardiac mortality (17).

Imaging and translational experiments provide complementary supportive evidence. Patients with elevated Lp(a) demonstrate increased uptake of 18F-sodium fluoride (18F-NaF), a biomarker marking active microcalcification; this phenomenon precedes progressive CT-detected valve calcium accumulation and rapid hemodynamic deterioration (23). *In vitro* cellular assays confirm Lp(a) drives osteogenic transformation of human VICs, whereas neutralizing OxPL substantially attenuates this mineralization phenotype (23). Recent pooled individual-participant data further verify that OxPL-apoB predicts prevalent and incident aortic valve calcium burden, as well as stenosis advancement—this predictive effect is particularly prominent among subjects with high Lp(a) (15). Collectively, these findings establish OxPL as a critical molecular bridge connecting systemic Lp(a) to localized valve mineralization. It should nevertheless be emphasized that OxPL biology is not confined to Lp(a): oxidized phospholipids also circulate on other apoB-containing lipoproteins and can be generated locally within the diseased valve, and the relative contribution of Lp(a)–bound versus total circulating OxPL to valvular calcification is not fully defined (12, 13). In terms of causal hierarchy, the most robust evidence identifies genetically elevated Lp(a) as the upstream determinant, whereas OxPL appear to mediate at least part of its valvular effect; whether lowering Lp(a)–associated OxPL alone, in the absence of other compositional changes, is sufficient to modify valve disease has not been established (13, 15).

### 3.2 The autotaxin–lysophosphatidic acid axis

Autotaxin (ATX) is a lysophospholipase D enzyme catalyzing the conversion of lysophosphatidylcholine into lysophosphatidic acid (LPA). ATX is highly enriched within circulating Lp(a) particles and can also be locally synthesized by diseased VICs within calcified aortic leaflets (24). In explanted calcified human valves, ATX colocalizes spatially with apo(a) and OxPL deposits. Preclinical functional studies reveal ATX-derived LPA activates nuclear factor-*κ*B (NF-*κ*B), interleukin-6 (IL-6) and bone morphogenetic protein (BMP) signaling cascades, thereby amplifying inflammatory responses and promoting hydroxyapatite crystal deposition within valve tissue (24). Circulating ATX enzymatic activity interacts synergistically with Lp(a) and OxPL concentrations to modulate individual CAVS risk (25). Although the ATX–LPA axis provides a compelling mechanistic link, the direct human evidence base is considerably smaller than that supporting the Lp(a)–CAVS association, deriving largely from a limited number of translational and small clinical studies (24, 25). Moreover, ATX and LPA are not specific to Lp(a): ATX is expressed in multiple tissues and circulates in association with several lipoproteins, and LPA signaling participates in broader lipid–inflammatory pathways. ATX should therefore be viewed as one component of a wider lipid–inflammator*y* axis implicated in CAVS rather than an Lp(a)-exclusive mechanism (24, 25). More recent experimental work supports this axis as an actionable target: autotaxin inhibition attenuated aortic valve calcification by suppressing inflammation-driven fibro-calcific remodeling of valvular interstitial cells, although these findings remain predominantly preclinical (26).

### 3.3 Endothelial dysfunction, inflammation, and osteogenic reprogramming

OxPL carried by Lp(a) upregulates endothelial adhesion molecules, accelerates monocyte recruitment, exacerbates intracellular oxidative stress and induces transcription of multiple pro-inflammatory genes (13, 20). Within valve leaflets, sustained inflammatory signaling triggers phenotypic switching of resident VICs toward myofibroblast-like and osteoblast-like cell lineages (5, 24). This cellular transition is accompanied by upregulated osteogenic mediators, augmented matrix vesicle secretion and progressive ECM mineralization (3, 5, 24). A self-amplifying pathogenic feedback loop thus forms: lipoprotein retention, local OxPL generation, persistent inflammation, interstitial fibrosis and progressive calcification. This cascade partially explains why therapeutic interventions initiated after massive valve calcification forms yield limited clinical benefits (3, 5, 22, 27, 28).

Although apo(a) shares structural homology with plasminogen, contemporary evidence suggests that impaired fibrinolysis is unlikely to be the primary mechanistic pathway linking Lp(a) to CAVS, although broader interactions of apo(a) with fibrinolytic and thrombotic pathways cannot be entirely excluded (9, 22, 28). The most consistent pathogenic model prioritizes four core processes: systemic OxPL transport via Lp(a), lipoprotein retention within valve tissue, ATX–LPA inflammatory signaling, and osteogenic reprogramming of interstitial cells (13, 15, 22, 24, 25, 28).

## 4 Evidence for causality, disease initiation, and progression

Genetic data deliver the most robust evidence supporting a causal relationship between Lp(a) and CAVS. LPA locus variants that raise circulating Lp(a) concentrations (including rs10455872 and rs3798220) correlate with aortic valve calcification and incident obstructive stenosis (6–8). Large population-based cohorts demonstrate dose-dependent associations between both genetically predicted and directly measured Lp(a) levels and clinical CAVS endpoints, including subsequent AVR (7–9). Since germline genotypes are fixed prior to disease onset, such genetic associations are far less susceptible to reverse causation bias compared with conventional observational cohort analyses.

Clinical cohort research confirms Lp(a) participates in both disease initiation and stenosis advancement in selected patient subgroups. Elevated Lp(a) correlates with prevalent and newly developed aortic valve calcium lesions (14–16), active microcalcification detected via 18F-NaF imaging, continuous CT calcium score elevation and accelerated hemodynamic worsening (15, 17, 23). A 2024 individual-participant meta-analysis pooling five longitudinal cohorts reported that patients within the highest Lp(a) tertile exhibited 41% faster peak aortic jet velocity progression and 57% steeper mean transvalvular gradient elevation relative to subjects in the lowest tertile (18). High Lp(a) retains independent predictive value for aortic stenosis events after adjustment for C-reactive protein, indicating its pathogenic pathway operates separately from systemic inflammatory status (19).

The strength of Lp(a)-related risk association varies with patient age: the genetic lipid pathway contributes a larger proportion of disease burden among younger patients. *Post-hoc* analyses from the ASTRONOMER trial demonstrated that excess risks linked to high Lp(a) and OxPL were markedly amplified in younger participants (17). Among elderly patients, pre-existing irreversible fibrosis, extensive macrocalcification, concurrent CKD, hypertension and other chronic comorbidities dilute the relative pathogenic contribution of Lp(a) (3, 16, 27). This stage-dependent pathogenic model carries critical therapeutic implications: Lp(a)-lowering interventions are predicted to achieve maximal efficacy during early calcific lesions or mild-to-moderate stenosis, before self-sustaining mineralization cascades dominate valve tissue pathology (3, 27, 28). These age- and stage-dependent dynamics carry direct implications for trial design: Lp(a)-lowering therapy may be most informative when tested in younger adults with early calcific lesions or mild-to-moderate stenosis rather than in unselected cohorts with advanced disease, and stratifying participants by age and baseline stage could increase the likelihood of detecting disease modification (3, 17, 18, 27, 28).

Not all published studies report identical correlative outcomes. For instance, one long-term population cohort observed Lp(a) correlates with incident aortic valve calcium onset yet fails to predict calcium score progression among patients with established baseline lesions (16). Heterogeneity across detection assay platforms, Lp(a) risk cutoffs, baseline stenosis severity, imaging endpoints, participant age ranges and follow-up durations complicates cross-study comparison (15, 16, 18, 27). Even so, consistent convergence across genetic, epidemiological, imaging and mechanistic datasets establishes Lp(a) as one of the most promising modifiable therapeutic targets for CAVS pathogenesis (9, 15, 18, 27, 28).

Table 1 summarizes the evidence linking Lp(a) across the distinct disease stages and clinical endpoints that define the CAVS spectrum. Because these endpoints are biologically and clinically different, the strength of the evidence for Lp(a) differs substantially between them. This heterogeneity is biologically meaningful: evidence is strongest for incident aortic valve calcification and the development of clinical aortic stenosis, intermediate for hemodynamic progression and the subsequent need for aortic valve replacement, and most limited for progression of established calcification and hard outcomes such as mortality (14–18).

**Table 1:** Summary of evidence linking lipoprotein(a) to different stages and clinical endpoints of calcific aortic valve disease.

Disease stage/endpoint	Strength of evidence for Lp(a)	Representative evidence	Interpretation and caveats
Incident aortic valve calcification	Strong	LPA variants and elevated Lp(a)/OxPL-apoB are linked to prevalent and newly detected leaflet calcification (6, 14–16).	Consistent across genetic and imaging datasets; most robustly demonstrated for disease onset.
Development of clinical aortic stenosis	Strong	Mendelian randomization and prospective cohorts show genetically predicted and directly measured Lp(a) predict incident obstructive aortic stenosis (7–9, 21).	Causal association supported at the population level; strongest causal evidence.
Progression of established calcification	Weak/heterogeneous	A long-term cohort found Lp(a) predicts onset but not progression of calcification (16); 18F-NaF studies link Lp(a) to active microcalcification (23).	Not uniformly demonstrated; depends on baseline valve stage and imaging endpoint.
Progression of transvalvular hemodynamic severity	Moderate	Higher Lp(a)/OxPL-apoB associate with faster peak jet velocity and gradient increase, especially in younger participants; a 2024 meta-analysis reported ∼41% faster jet-velocity progression (17, 18).	Seen in selected (younger, mild-to-moderate) cohorts, not in unselected populations.
Need for aortic valve replacement (AVR/TAVR)	Moderate	Higher Lp(a) associates with increased risk of subsequent AVR in cohorts and *post-hoc* analyses (7, 8, 17).	Moderate; not established within a dedicated CAVS trial setting.
Mortality (all-cause/cardiac)	Limited	Evidence for all-cause or cardiac mortality is limited, drawn mainly from observational cohorts (17).	Weakest endpoint; confounded by competing cardiovascular risk.

AS, aortic stenosis; AVR, aortic valve replacement; CAVS, calcific aortic valve stenosis; CT, computed tomography; Lp(a), lipoprotein(a); MR, Mendelian randomization; OxPL, oxidized phospholipids; TAVR, transcatheter aortic valve replacement; VIC, valve interstitial cell; 18F-NaF, 18F-sodium fluoride.

The strength of evidence is a qualitative assessment of the consistency and quality of genetic, imaging and clinical data supporting an association between Lp(a) and each stage or endpoint; it does not represent a quantified effect size and should be interpreted alongside the detailed discussion in the text.

Figure 1 illustrates the conceptual disease trajectory linking elevated Lp(a) to hemodynamically significant aortic stenosis and indicates the stage at which Lp(a)-lowering therapy is hypothesized to exert its greatest effect.

**Figure 1:**
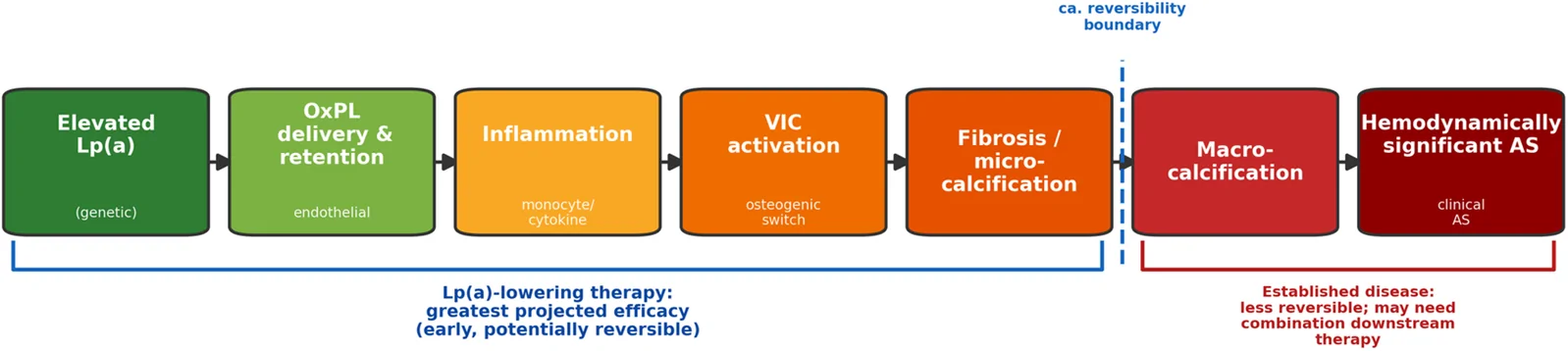
Conceptual model of Lp(a)-driven calcific aortic valve disease progression, from elevated circulating Lp(a) through OxPL delivery and retention, inflammation, valvular interstitial cell (VIC) activation and microcalcification to macrocalcification and hemodynamically significant aortic stenosis. The dashed vertical line denotes the approximate boundary between potentially reversible early disease and less reversible established disease. The blue bracket indicates the stage at which Lp(a)-lowering therapy is projected to be most efficacious; the red bracket denotes established disease that may require combination downstream therapy.

## 5 Clinical implications and risk stratification

At present, Lp(a) testing among CAVS patients should be regarded as a risk-enhancing biomarker rather than a standalone indicator guiding therapeutic decision-making (9–11, 27). Consistent with contemporary cardiovascular prevention guidelines, one-time Lp(a) measurement is recommended primarily for global ASCVD risk stratification; in patients with CAVS the result should be interpreted within this broader cardiovascular risk framework rather than as a CAVS-specific diagnostic or management tool. Within this context, Lp(a) measurement may be particularly informative for four patient subgroups: individuals with premature valve calcification, patients with a family history of CAVS or premature atherosclerotic cardiovascular disease (ASCVD), subjects presenting unexpectedly rapid stenosis progression, and patients with residual cardiovascular risk burden despite optimized LDL-C control (9–11). Real-world data additionally show a high burden of atherosclerotic cardiovascular disease and suboptimal LDL-C target attainment among subjects with markedly elevated Lp(a) (29).

Identification of elevated Lp(a) necessitates comprehensive optimization of all modifiable cardiovascular risk factors. Clinical interventions include smoking cessation, standardized blood pressure management, glycemic control for diabetes mellitus, weight optimization and regular physical activity, alongside intensive LDL-C lowering aligned with individual overall ASCVD risk stratification (9–11). Though these lifestyle and lipid strategies cannot substantially reduce genetically determined Lp(a) concentrations, they effectively mitigate total cardiovascular risk exposure for patients carrying high Lp(a) levels (9–11).

No validated risk algorithm based solely on Lp(a) concentrations currently dictates timing for echocardiographic surveillance or valve replacement intervention. Clinical monitoring should remain guided by guideline-recommended assessments covering symptomatic status, aortic jet velocity, mean transvalvular gradient, effective valve orifice area, left ventricular functional response and exercise stress testing when clinically appropriate (2). CT aortic valve calcium scoring serves as a complementary imaging modality alongside echocardiography for selected patients, offering a reproducible quantitative metric for total calcific burden (2, 30). Lp(a) measurement may eventually facilitate enrichment of clinical trial cohorts or identify patients requiring intensified follow-up protocols, yet prospective validation studies are still required to confirm this clinical utility (18, 27, 28).

## 6 Therapeutic strategies

### 6.1 Conventional lipid-lowering therapy

Multiple randomized controlled trials evaluating intensive statin-centered lipid-lowering regimens—including SALTIRE, SEAS and ASTRONOMER—failed to slow progression of established CAVS lesions (31–33). Such results do not negate lipid pathway involvement during the initial disease priming phase; instead, they demonstrate isolated LDL-C reduction exerts limited efficacy after extensive valve calcification has developed (3, 5, 22, 27, 28). Statins remain first-line therapy for standard ASCVD primary and secondary prevention, yet clinicians should not prescribe statins specifically to modify CAVS disease trajectory (2, 3).

Monoclonal antibodies targeting PCSK9 enhance LDL receptor recycling and deliver a moderate 20%–30% reduction in circulating Lp(a), in addition to profound LDL-C lowering effects (9–11, 34). An exploratory *post-hoc* analysis from the FOURIER trial indicated baseline Lp(a) predicted incident aortic stenosis events, while evolocumab yielded preliminary suggestive risk reduction signals after the first year of treatment (34). However, this analysis carried inherent limitations: it was not prospectively designed for CAVS endpoints, event counts remained low, and the profound concomitant reduction in LDL-C precludes attributing any observed signal specifically to Lp(a) lowering. Critically, PCSK9 inhibition is not an Lp(a)-specific therapy, so these exploratory findings should not be interpreted as evidence that Lp(a) lowering modifies CAVS. Dedicated randomized trials focusing on CAVS progression are still required to confirm any disease-modifying efficacy.

### 6.2 Lipoprotein apheresis

Lipoprotein apheresis removes apoB-containing lipoproteins extracorporeally and acutely reduces Lp(a) concentrations by approximately 60%–75%, yet circulating Lp(a) rebounds rapidly between treatment sessions (9–11, 35). This intervention consumes substantial medical resources and is generally reserved for highly selected patients with extremely elevated Lp(a) and progressive ASCVD despite maximal guideline-directed medical therapy (10, 11). Small randomized and observational studies report improved myocardial perfusion and alleviated angina symptoms among patients with refractory coronary artery disease (35), but robust clinical evidence confirming apheresis slows CAVS progression remains absent.

### 6.3 RNA-targeted therapies

Pelacarsen is a hepatocyte-directed antisense oligonucleotide (ASO) suppressing hepatic apo(a) biosynthesis. Phase 2 clinical trials observed dose-dependent Lp(a) reduction reaching up to 80% among participants with pre-existing cardiovascular disease (36). Olpasiran, a N-acetylgalactosamine-conjugated small interfering RNA (siRNA), achieved Lp(a) suppression exceeding 95% at high doses in the OCEAN(a)-DOSE trial (37). Additional siRNA agents deliver similarly profound and durable lipid-lowering effects: lepodisiran sustains stable Lp(a) reduction with infrequent administration intervals, and zerlasiran lowers time-averaged Lp(a) levels by over 80% in phase 2 investigations (38, 39). Although these RNA therapeutics establish the technical feasibility of profound Lp(a) suppression, no trial has yet validated that such biomarker reduction translates into attenuated CAVS progression. Moreover, it remains uncertain whether any clinical benefit will depend exclusively on the magnitude of Lp(a) lowering or on associated changes in OxPL, inflammation and other downstream pathways. A recently published comprehensive review summarizes these unresolved questions and the current state of targeted Lp(a)–lowering drug development (40).

### 6.4 Oral Lp(a) assembly inhibitors

Muvalaplin represents an oral small-molecule agent disrupting noncovalent intermolecular interactions required for intact Lp(a) particle assembly. A phase 2 randomized trial demonstrated daily oral muvalaplin induced dose-dependent Lp(a) reduction up to roughly 86% measured via intact-Lp(a) assays, with acceptable overall tolerability over a 12-week treatment period (41). If long-term safety and clinical efficacy are validated in subsequent trials, oral targeted therapy will greatly expand clinical accessibility to Lp(a)-lowering interventions.

### 6.5 Aortic-stenosis-specific clinical testing

Sustained biomarker reduction alone cannot confirm a therapeutic agent's capacity to reverse fibrocalcific valve pathology. Two critical determinants govern treatment efficacy: sufficient early intervention timing and prolonged consistent drug exposure to alter progressive valve remodeling (3, 18, 27, 28). The ongoing randomized, double-blind, placebo-controlled Lp(a)FRONTIERS CAVS trial (NCT05646381) evaluates monthly pelacarsen administration in an estimated 502 CAVS patients; participant recruitment remains active, with primary trial completion projected for March 2030 (42). This trial constitutes the first prospective dedicated investigation testing whether selective Lp(a) suppression modifies CAVS disease trajectory, rendering it a landmark study within this research field. Table 2 systematically summarizes molecular mechanisms, magnitude of Lp(a) reduction, and CAVS-specific clinical evidence for all abovementioned therapeutic pipelines.

**Table 2:** Current and emerging Lp(a)-lowering strategies and their relevance to calcific aortic stenosis.

Therapy	Mechanism	Typical Lp(a) effect	Evidence relevant to CAVS	Current interpretation
Statins	HMG-CoA reductase inhibition; major LDL-C lowering	No meaningful reduction; Lp(a) may increase modestly (9, 10, 22)	SALTIRE, SEAS, and ASTRONOMER did not slow established CAVS (31–33)	Use for standard ASCVD indications, not as CAVS-modifying therapy
PCSK9 monoclonal antibodies	Increase LDL-receptor recycling	Approximately 20%–30% reduction (9–11, 34)	Exploratory FOURIER signal; no dedicated positive CAVS trial (34)	Hypothesis-generating; not proof of CAVS disease modification
Lipoprotein apheresis	Extracorporeal removal of apoB-containing lipoproteins	Acute reduction of approximately 60%–75% (9–11, 35)	No CAVS-specific outcome evidence; randomized evidence is limited to refractory coronary disease (35)	Reserved for selected patients with progressive ASCVD and very high Lp(a)
Pelacarsen	Antisense oligonucleotide targeting apo(a) mRNA	Up to approximately 80% reduction (36)	Dedicated Lp(a)FRONTIERS CAVS phase 2 trial is recruiting (42)	Only targeted Lp(a)-lowering agent currently being tested in a dedicated CAVS trial
Olpasiran	siRNA targeting apo(a) synthesis	More than 95% reduction at higher phase 2 doses (37)	No completed CAVS-specific outcome trial (37)	Potent investigational therapy; CAVS efficacy remains unproven
Lepodisiran	Long-duration siRNA targeting apo(a) synthesis	More than 90% reduction with prolonged effects in phase 2 testing (38)	No completed CAVS-specific outcome trial (38)	Potent, infrequently dosed investigational therapy; CAVS efficacy remains unproven
Zerlasiran	siRNA targeting apo(a) synthesis	More than 80% time-averaged reduction in phase 2 testing (39)	No completed CAVS-specific outcome trial (39)	Investigational therapy; CAVS efficacy remains unproven
Muvalaplin	Oral inhibitor of Lp(a) particle assembly	Up to approximately 86% reduction by intact-Lp(a) assay (41)	No completed CAVS-specific outcome trial (41)	First oral targeted approach in clinical development; CAVS efficacy remains unproven

ASCVD, atherosclerotic cardiovascular disease; CAVS, calcific aortic valve stenosis; Lp(a), lipoprotein(a); PCSK9, proprotein convertase subtilisin/kexin type 9; siRNA, small interfering RNA.

Reported Lp(a) reductions are approximate and may vary according to dose, dosing interval, assay method, treatment duration, and study population. CAVS-specific trial status is current to 25 July 2026; among targeted Lp(a)-lowering agents, pelacarsen is the only agent currently being evaluated in a dedicated randomized CAVS trial (42).

## 7 Knowledge gaps and future directions

Multiple unresolved scientific and clinical uncertainties remain in this research field, categorized into three core dimensions: optimal intervention timing, required magnitude/duration of Lp(a) suppression, and ideal predictive biomarkers. First, the optimal disease stage for initiating Lp(a)-targeted therapy remains undefined. Advanced CAVS with extensive macrocalcification exhibits limited biological reversibility relative to early microcalcific lesions, making precise patient subgroup stratification indispensable for future trials (3, 5, 27, 28). Second, researchers have not established the minimal Lp(a) reduction magnitude and sustained treatment duration required to improve hard clinical valve endpoints (18, 27, 28). Third, it remains unclear which biomarker—single indicator (Lp(a), OxPL-apoB, ATX activity, CT calcium score, 18F-NaF uptake) or multimodal combined panel—best identifies patients responsive to Lp(a)-lowering treatment (15, 23, 25, 30, 43).

Upcoming clinical trials should standardize Lp(a) detection assays and reporting units, recruit multi-ethnic participant cohorts, differentiate research endpoints between disease initiation and hemodynamic stenosis progression, and integrate both imaging surrogate markers and hard clinical outcomes (9–11, 18). Serial CT calcium scoring and 18F-NaF positron-emission tomography (PET) can detect therapeutic biological effects far earlier than clinical endpoints such as AVR or all-cause mortality (30, 43). Embedded mechanistic substudies within large outcome trials are needed to clarify whether Lp(a) lowering simultaneously reduces tissue OxPL burden, suppresses ATX–LPA signaling and inhibits VIC osteogenic transformation within aortic valve leaflets (15, 23–25).

Multimodal combination therapeutic regimens are likely to be required for comprehensive CAVS management, because a single upstream target is unlikely to reverse established disease. Within this framework, Lp(a)–targeted therapy functions primarily as an upstream preventive intervention aimed at interrupting OxPL delivery, inflammation and early osteogenic reprogramming during the initial disease priming phase. In contrast, established fibrocalcific lesions with self-sustaining mineralization may require additional strategies targeting downstream inflammation, interstitial fibrosis, osteogenic signaling, mineralization and ATX/LPA receptor signaling (3, 5, 24, 28). Such combinatorial approaches demand rigorous long-term safety evaluation, given the ubiquitous physiological functions of mineral metabolism and tissue wound-repair pathways across multiple organ systems.

## 8 Conclusions

Genetic, translational and clinical evidence supports a causal role for Lp(a) in the initiation, and an association with the advancement, of calcific aortic valve disease (6–9, 18, 19); this causal relationship is most firmly established for disease initiation rather than for progression of established severe stenosis. Its valve-damaging effects are primarily mediated through two core processes: Lp(a) retention within valve leaflets and systemic OxPL delivery, which sequentially activate inflammatory cascades and the ATX–LPA signaling axis to trigger VIC osteogenic transformation and tissue mineralization (12, 13, 15, 17, 20, 21, 23–25). Elevated Lp(a) correlates with disease onset, accelerated stenosis advancement in selected cohorts and adverse valve-related clinical outcomes, yet its predictive performance fluctuates according to patient age, baseline disease stage and selected research endpoints (14–19).

Current clinical management integrates guideline-standardized CAVS surveillance protocols with intensive control of all modifiable cardiovascular risk factors (2, 9–11). No Lp(a)-targeted therapy has yet been validated to prevent or slow CAVS progression, and surgical/TAVR valve replacement remains the definitive curative intervention for patients with severe symptomatic stenosis (2, 3). The advent of potent ASO, siRNA and oral Lp(a)-suppressing agents—alongside the landmark dedicated Lp(a)FRONTIERS CAVS trial—delivers the first realistic opportunity to determine whether interventions targeting this genetically regulated lipid pathway can achieve disease-modifying effects in CAVS patients (36–39, 41, 42).
